# Considerations on the implementation of DCT: a SCAT-based analysis of fact-finding interviews in Europe and the United States, with implications for regions newly adopting DCT, including Japan

**DOI:** 10.3389/fmed.2025.1521135

**Published:** 2025-10-31

**Authors:** Hiroyuki Taruno, Koji Ikeda, Marika Oikawa, Takahiro Sato, Yuka Suzuki, Mayumi Shikano

**Affiliations:** ^1^Cancer Institute Hospital of Japanese Foundation for Cancer Research, Koto-ku, Japan; ^2^Develop Promotion, Clinical Research Innovation and Education Centre, Tohoku University Hospital, Sendai, Japan; ^3^International Affairs, Clinical Research Innovation and Education Centre, Tohoku University Hospital, Sendai, Japan; ^4^Faculty of Pharmaceutical Sciences, Tokyo University of Science, Noda, Japan

**Keywords:** internet of things, electronic consent, decentralized clinical trials, partner healthcare organizations, steps for coding and theorization

## Abstract

**Introduction:**

Decentralized clinical trials (DCT) are becoming more common. In regions where DCT will be widely adopted, including Japan, issues in implementing DCT must be identified and addressed.

**Materials and methods:**

In this study, we interviewed the clinical development staff at pharmaceutical and medical device companies, or clinical research organizations in Europe and the United States and then analyzed their information to understand the transition to and current status of DCT from a practical perspective. Steps for Coding and Theorization qualitative data analysis was used.

**Results:**

DCT, along with the rapid digitisation of medical care, occurred in some institutions because of the novel coronavirus disease pandemic. Our results confirmed that introducing DCT allowed patients who would otherwise have struggled to participate in traditional trial formats to have easier access to clinical trials, allowing them to experience new treatments and reducing the inconvenience of travel burdens and waiting times for patients who previously had to travel long distances to medical institutions to participate in clinical trials. However, introducing new DCT can be challenging for several reasons, including local culture, regulations regarding home and telemedicine, online sharing of medical record information with trial personnel, the development of Internet of Things infrastructure, information technology literacy of trial personnel and subjects, and the associated costs.

**Discussion:**

We identified specific issues common to medical devices and pharmaceutical clinical trials. In addition, the experiences of those in charge were used to identify specific issues in the DCT introduction phase. Information on the latest overseas DCT methods, such as patient neighborhood institutions and remote services that use ambulances, mobile vans, tents, supermarkets, and pharmacies to replace implementing medical institutions, was obtained. Currently, Japan lags behind Europe and the United States in terms of DCT diffusion. However, we hope to resolve many of the aforementioned issues in the future to actively introduce DCT in Japan, thereby preventing Japan from being left behind in international joint trials. Furthermore, this study’s findings will be of significant value to countries and regions that are striving to fully adopt DCT.

## 1 Introduction

The impact of the novel coronavirus disease (COVID-19) pandemic restricted access to medical facilities, resulting in the promotion of decentralized clinical trial (DCT), in which a few (hybrid) or all (site-less) clinical trial-related activities are conducted using the Internet of Things (IoT) at locations other than traditional medical facilities (1–3); these facilities are primarily located in Europe and the United States. A DCT is a form of clinical trial that does not require participants to visit a clinical trial site; instead, they participate at a medical facility near their home or through in-home healthcare.

Many of the components or modalities used to conduct a DCT existed before the COVID-19 pandemic, including wearable devices, video calls, shared cloud-based electronic medical records, follow-up checkups at home visits (such as blood sampling and electrocardiogram), telemedicine, remote accounting systems, and systems that can centralize each solution. The development of Electronic Informed Consent (eConsent) and electronic patient-reported outcome (ePRO), among other electronic consent solutions, was important.

**Figure 1:**
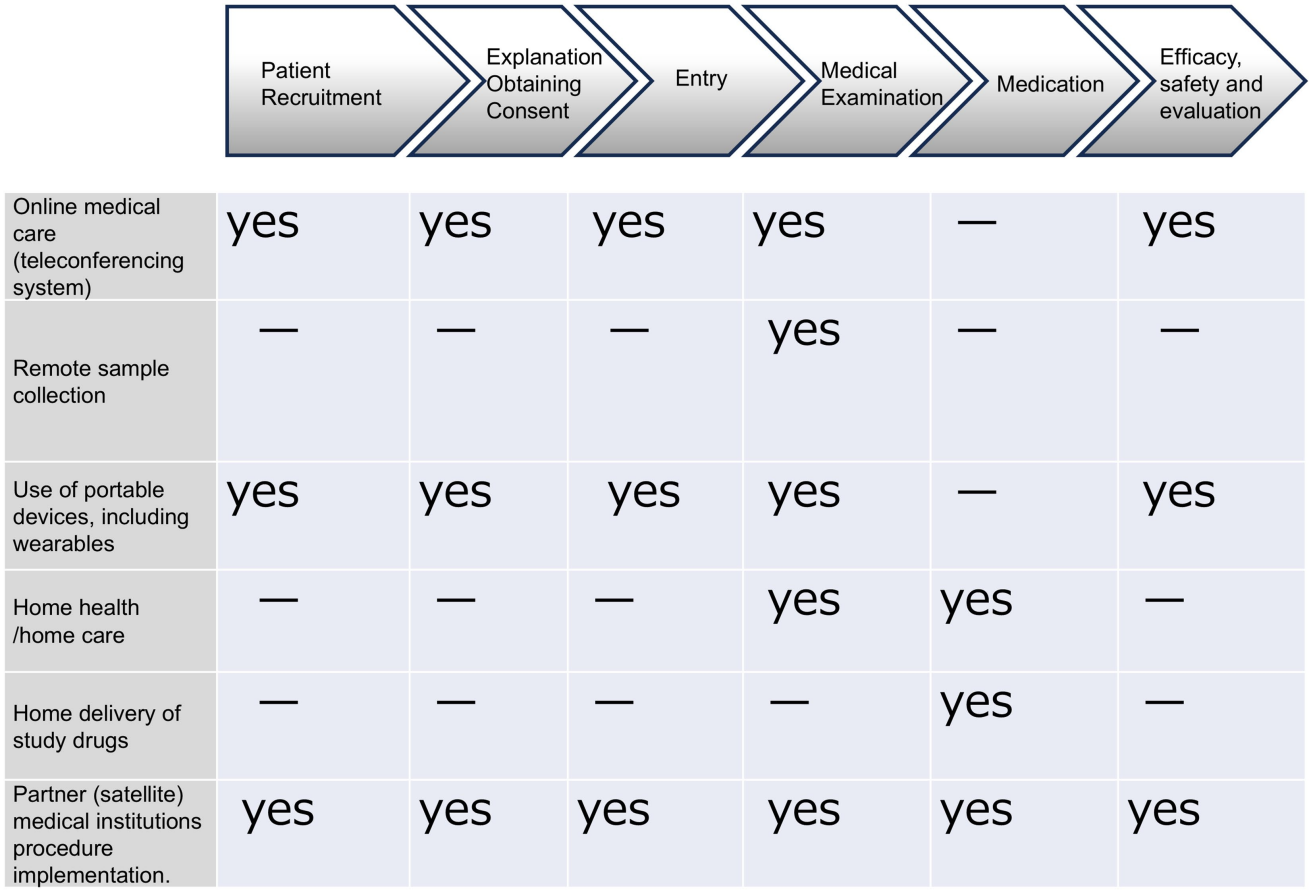
In DCT, various components (modalities) are being considered for practical application and efficiency improvement. Clinical trials are conducted remotely using IT and systems outside of medical institutions. There are six main components (modalities): online medical care (teleconferencing systems), remote sample collection, the use of portable devices, including wearables, home health/home care, home delivery of study drugs, and partner medical institutions for medical treatment. Each component (modality) is utilized according to the stage of the clinical trial.

As Figure 1 shows, these components and modalities can be divided into six categories: online medical care (teleconferencing system); remote blood collection; use of portable devices, including wearables; home health/home care and home delivery of study drugs; and partner (satellite) medical institutions procedure implementation.

Even before the COVID-19 pandemic, regulatory authorities in Europe and the United States published guidelines for clinical trial-related IoT (4–7) intended to streamline clinical trial promotion, including data collection, which contributed to the spread of DCT.

Because the development of new pharmaceuticals and medical devices is increasing the number of clinical trials conducted globally, establishing DCT environments in Europe and the United States (8–12) is expected to lead to an increase in global developments that use DCT in the near future. Although several regulatory documents regarding clinical trial-related IoT (13–16) have been published or are being drafted in Japan at the time of 2021, other critical DCT guidance documents will likely become available, including partner medical institutions, IT platforms, and investigational new drug delivery. At the same time, stakeholders in clinical trials in Japan must understand the challenges involved in DCT implementation. However, information on this topic is not well organized.

We surveyed existing literature on DCT. Before 2022, there were numerous problematic and negative opinions regarding DCT (17–19). However, after 2022, analyses emerged concerning the regulations surrounding DCT, alongside more positive perspectives on various components and the clinical trials that were actively being conducted.

In this study, we conducted interviews with practitioners who have extensive experience in implementing DCT, with the aim of organizing practical information on DCT in Europe and the United States, regions where the global pandemic has driven significant changes. The interviews also explored challenges and corresponding responses encountered in promoting the broader adoption of DCT. The results of this survey are intended to serve as a reference for potential obstacles and countermeasures in regions, including Japan, that have limited experience with DCT and are seeking to further promote its use. This paper does not compare the current status of DCT in Europe, the United States, and Japan, nor does it introduce the latest trends in DCT in Japan or the opinions of related parties.

The information obtained from the interviews was not derived from quantitative research but was instead analyzed using SCAT (Steps for Coding and Theorization) (20). In qualitative research, particularly studies involving interviews that may include confidential information, it is essential to gain a nuanced understanding of the perspectives of a small number of participants. In this study, we specifically sought the views of individuals directly involved in the practical aspects of DCT implementation. Although the interviews were conducted individually, SCAT analysis was employed to reduce bias and ensure the extraction of objective insights. SCAT is frequently used to explore emerging concepts and to derive subjective interpretations from a small pool of experts with specialized knowledge. However, because qualitative research often incorporates subjective elements, it can risk becoming highly arbitrary. To address this, Supplementary Datasheet 1, Supplementary Tables 1-5 document the analysis process in an objective and transparent manner. This structure compels analysts to reflect critically on their interpretations, thereby enhancing the validity of the findings and making the method well-suited for collaborative analysis. In addition, to confirm the reliability of the survey results, a member check was conducted, and interview participants confirmed that their perspectives were accurately reflected in the coded transcripts. In this study, multiple authors conducted the analysis, which helped minimize inter-rater variability and reduce potential bias.

SCAT analysis offers several key advantages: it is accessible to researchers new to qualitative analysis; it is effective for small-scale data sets such as interviews and questionnaires; it maintains objectivity and transparency through step-by-step documentation using Microsoft Excel; and it facilitates the construction of a coherent analytical narrative.

The insights gained from this study provide valuable guidance for countries and regions seeking to implement DCT more comprehensively.

## 2 Materials and methods

### 2.1 Interviews

In-depth interviews were conducted with 12 clinical development managers with extensive experience in DCT in Europe and the United States. These managers had worked for five global companies, including two medical device companies, two pharmaceutical companies, and one clinical research organizations (CRO). The interviews were conducted online in November and December 2021 or January 2023.

Two global pharmaceutical companies were selected for interviews based on their extensive experience conducting DCTs in Europe and the United States, as well as their significant involvement in international clinical trials that include Japan. Additionally, two medical device companies and one CRO were selected because they are among the few organizations with experience in conducting international medical device trials in both Japan and the United States through the *Harmonisation by Doing* (HBD) initiative.

HBD is a project that was launched when international joint clinical trials for medical devices were still relatively limited. Its goal was to harmonize regulatory processes for medical devices between Japan and the United States through practical collaboration among industry, academia, and government. The initiative specifically focused on clinical trials and regulatory review procedures.

Interview participants were selected at random based on recommendations from HBD or internal recommendations from each participating organization. Eligible participants were those with substantial experience in DCT-related clinical trials. Supplementary Datasheet 2 was shared with each company in advance to guide participant selection, along with a request to nominate individuals capable of responding to the listed questions based on their practical experience with DCT implementation. HBD is merely a selection criterion for this study, and improving the service quality of HBD itself is not the purpose of this paper.

The interview guide, which outlines the key discussion points for the interviews, is shown in Supplementary Datasheet 3, Figure 1. The questionnaire sent to each company in advance for interviewee selection is provided in Supplementary Datasheet 2.

### 2.2 SCAT analysis

Because each interviewee’s comments or responses could potentially be disadvantageous to them or their companies, all interview content was integrated before the SCAT analysis. As shown in Supplementary Datasheet 1, Supplementary Tables 1-5 document the interview transcripts were segmented and written in Microsoft Excel; Steps 2–6 were then carried out.

Step 1: Write down the words and phrases that are most noteworthy or important in the text.

Step 2: Paraphrase words and phrases in the text.

Step 3: Fill in extra-textual concepts and phrases that explain Step 2.

Step 4: Describe the themes and concepts derived from Steps 2 and 3.

Step 5: Input questions and issues to be pursued during the analysis.

Storylines were created considering the context of the sentences before, after, left, and right, based on the themes and concepts observed in Step 4. The storyline was organized based on the themes/constitutive concepts observed in Step 4 and recontextualised the interview content. Significant theoretical explanations were extracted from the storyline, and questions and issues derived from the storyline and theoretical explanation were entered as issues to be pursued to clarify the issues and facilitate consideration.

## 3 Results

Twelve clinical development managers with experience in conducting DCT from five companies with affiliates outside Japan were interviewed. The total interview duration was 5 h and 10 min. The storyline created by the SCAT analysis is shown below. Refer to Supplementary Datasheet 1, Supplementary Table 5 for details of the theoretical explanations and issues to be pursued.

Themes were determined by the authors prior to the interviews. SCAT analysis was conducted on all seven themes, and the authors selected four themes that he deemed important, for which responses were obtained from all participants.

### 3.1 The storyline

The results of the SCAT analysis typically include a description of the storyline. Information regarding the implementation status in the United States before and after the outbreak of COVID-19 was specifically obtained from medical device companies.

#### 3.1.1 DCT implementation in Europe and the United States before the COVID-19 pandemic

Before the COVID-19 pandemic, DCT was a new concept. Medical facilities and physicians were originally concerned about introducing the trial method, as they had little experience in operating DCT. A few physicians were willing to adopt the IoT approach to obtain consent, monitor data, and other aspects of patient care; however, this willingness was not universal. Ethics review committees require a detailed explanation of how the DCT would operate, including its appropriateness, and committee members express concerns about whether data could be accessed from outside the institution (17, 18). External access to medical records is possible at certain institutes. Interviewees mentioned concerns regarding how stakeholders, including sponsors, facilities, and partner institutions, would share the costs of introducing DCT. Furthermore, many factors, including the lack of robust systems at medical facilities, have inhibited the introduction of DCT (Supplementary Datasheet 1, Supplementary Table 1, Step 4. 1-7).

In Europe, nurses are licensed by their country, and in the United States, they are licensed by their state. In both Europe and the United States, nurses perform their duties under the direction of a physician. This study highlights the importance of the medical environment, including regulations, digital tools, and the establishment of decentralized procedures in clinical trials.

#### 3.1.2 DCT implementation in Europe and the United States after the COVID-19 pandemic

Since January 2020, the global spread of COVID-19 has significantly impeded the promotion of clinical trials and submission of product applications for approval. The lockdowns implemented to try and control the spread of COVID-19 prevented patients from visiting hospitals, forcing hospitals to continue their clinical trials by implementing DCT. As DCT systems were developed, institutional implementation frameworks and procedures were created, and vendors offering DCT systems emerged. Remote monitoring is now a viable option in almost all facilities. Consequently, the mindset for how clinical trials can be conducted has changed with the implementation of DCT, and positive perceptions of DCT have accelerated their introduction.

Because trials need to be conducted without monitors visiting the facility, most facilities in the United States now have external access to data. In addition, medical device training can now be performed using virtual reality and other technologies outside medical institutions (Supplementary Datasheet, Supplementary Table 2, Step 4. 8-13).

#### 3.1.3 The advantages and disadvantages of DCT implementation

The introduction of the DCT (Supplementary Datasheet, Supplementary Table 3, Step 4. 14-17) offers several advantages, including accessibility to patients in remote areas who struggle to participate in conventional clinical trials. This approach facilitates greater participation in clinical trials, thereby expanding the diversity of participants.

DCT can reduce patient inconvenience and provide a beneficial environment. For example, instead of requiring hours of observation, the core facility can remotely monitor patients during visits and acquire real-time data to confirm patient safety through video conferences. Thus, the burden of patient travel and waiting time can be reduced.

DCT facilitated follow-up and improved patient compliance. Implementing remote clinical trial procedures has enhanced efficiency and flexibility in many ways, including how clinical trials are conducted. DCT allows data to be collected outside visits, which may reduce the amount of missing data and improve clinical trial quality.

However, there are several disadvantages to introducing the DCT (Supplementary Datasheet 1, Supplementary Table 3 Step 4. 18-22).

First, all physicians, medical staff, and patients involved in clinical trials must demonstrate an affinity for digital technology. For example, a lack of access to technology or the inability to utilize it may restrict participation in a DCT.

Second, collecting patient information through wearables and other devices requires patients to understand the process and be actively involved in clinical trials. Without patient understanding and cooperation, verifying whether the entered data are accurate and ensuring their integrity and reliability is difficult. In addition, the possibility of continuous data collection via devices may increase noise.

Third, there are cases where data could be affected by the testing equipment and conditions, and the condition settings in the DCT need to be studied in detail. Because there are multiple facilities, testing and other conditions must be unified.

In the near future, the costs will increase with the development of new systems and structures.

We were able to obtain information regarding the advantages and disadvantages of DCT implementation from all companies.

#### 3.1.4 Challenges in introducing the DCT

The first challenge in introducing DCT is the cultural awareness of the region where it is being implemented. For instance, in Japan, direct communication with primary care physicians is important for patients, while patients in China may not favor home care or telemedicine; thus, healthcare professionals and patients might be hesitant to choose DCT. A conservative disposition or reluctance to embrace change is another obstacle. The prevailing culture among healthcare providers suggests that face-to-face clinical trials should be conducted. In other words, consensus and a shared mindset among all stakeholders are crucial for the successful implementation of the DCT (Supplementary Datasheet 1, Supplementary Table 4, Step 4. 23, 24). This issue has been recognized by pharmaceutical companies with affiliates outside Japan.

Second, numerous practical regulatory differences must be addressed on a country-by-country basis, such as regulations concerning mobile nurses and home health nursing, which require resources and can hinder DCT implementation (21). For example, at the time of the interviews, Canadian regulations did not permit mobile nurses, whereas French regulations prohibited the use of eConsent due to privacy concerns (Supplementary Datasheet 1, Supplementary Table 4, Step 4. 25). All the companies interviewed mentioned this issue.

Third, IT literacy, often referred to as the digital divide, is a significant concern. All study personnel, including patients, physicians, and staff, must adapt to the IoT technology. Their familiarity with IoT and the age of the patients determine their ability to participate in DCT. Trials involving older adults, in particular, require attentive technical support, which may place an additional burden on clinical research coordinators (CRCs). However, there are currently insufficient measures to address this increased workload. Furthermore, educating patients and conducting periodic checks are essential to ensure that the data entered by the patients themselves are of high quality. Securing digital human resources from participating medical institutions is crucial (Supplementary Datasheet 1, Supplementary Table 4, Step 4. 26). We were able to obtain this assignment from four of the five companies.

Fourth, although various data transmission methods and platforms exist for data acquisition, it is critical to ensure that personal information and privacy are protected and that data are validated through a compliant system. The challenge lies in protecting patients’ personal information while improving the quality of clinical trials at the facility level. Consequently, small-scale pilot studies, similar to those conducted in the United States, should be conducted in Japan to determine the optimal method of application (Supplementary Datasheet 1, Supplementary Table 4, Step 4. 27). All the companies interviewed mentioned this issue.

Fifth, when introducing DCT, it is necessary to create contracts and procedure manuals for each medical institution, which can be costly and personnel intensive. Furthermore, regulators such as the FDA and EMA require sponsors to verify that study personnel, including contractors, are qualified to perform the DCT. This entails complicated procedures, including vendor monitoring, and increases the burden on the support departments, CRO, and CRC (Supplementary Datasheet 1, Supplementary Table 4, Step 4. 29). We were able to obtain this information from four of the five companies.

Finally, the traditional study protocol must be significantly modified, which delays the finalization of the protocol and initiation of clinical trials. Those responsible for conducting clinical trials, including physicians and sponsors who are accustomed to conventional study protocols, often believe that conventional methods lead to the early termination of clinical trials. This issue has been recognized by pharmaceutical companies with affiliates outside Japan.

Moreover, regional disparities may deter the implementation of DCT. Healthcare professionals may possess a conservative mindset, leading to reluctance to accept DCT. They may perceive the adoption of dynamic and innovative changes as risky (Supplementary Datasheet, Supplementary Table 4, Step 4. 28). This issue has been recognized by pharmaceutical companies with affiliates outside Japan.

## 4 Discussion

In this study, we used SCAT analysis of interviews with clinical development practitioners in pharmaceutical and medical device companies and CROs in Europe and the United States to understand the current situation of DCT. In particular, we compared the situation before and after the COVID-19 pandemic, which was a major catalyst for the spread of DCT, to clarify the challenges facing regions trying to promote the introduction of DCT, including Japan. Our results provide useful information for the global promotion of DCT.

### 4.1 SCAT analysis

As previously noted, qualitative research such as interview-based studies requires a detailed understanding of interviewees’ perspectives, accurate capture of their opinions, and contextual presentation of their experiences, particularly when dealing with confidential data. The primary qualitative methods include thematic analysis (TA) (22) and SCAT (20). In TA, data are thoroughly reviewed, codes are assigned, and similar codes are grouped into themes that facilitate comprehensive understanding. However, TA results may vary depending on the analyst’s interpretation.

SCAT analysis, developed by Otani (20), has previously been applied to investigate factors causing Japanese patients with type 2 diabetes to hesitate in starting insulin therapy (23) and to analyze interviews conducted by primary care physicians abroad (24). We selected SCAT for this study because it is considered more objective than TA and minimizes the influence of analyst subjectivity. To address common challenges faced by beginners in SCAT analysis, such as the order of analysis and rephrasing of expressions, as well as inconsistencies in coding, thorough preparation and meticulous transcription of interview content were conducted.

### 4.2 Major significance of the introduction of DCT

The storylines from this interview (Supplementary Datasheet 1, Supplementary Table 4, Step 4. 14, 15) indicates that the major significance of introducing DCT is that patients who would have had difficulty participating in conventional clinical trials gain the ability to access trials more easily and can benefit from new treatments. The inconvenience of a long travel time is eliminated by removing the need to visit the facility. While conventional clinical trials that are conducted by gathering patients at a facility are physician- and healthcare professional-centered, DCT is a patient-centered method that can be further promoted in the future.

However, if the physicians and medical staff involved struggle with patient-centered DCT, their introduction and dissemination are unlikely, unless those difficulties are solved.

### 4.3 Regional and cultural differences and regulations regarding DCT

In Europe and the United States, the COVID-19 lockdown forced most clinical trials to be completely halted because trial participants could not come to institutions. The development of new drugs was halted for pharmaceutical companies and other organizations, which was extremely serious. Consequently, expectations and demand for DCT utilizing telemedicine have increased rapidly, and many DCT have been implemented at an accelerated pace. The interviews showed that other than in France and Canada, there were no regulations in Europe or America to prevent the implementation of DCT before the COVID-19 pandemic.

However, in Japan, under the COVID-19 emergency declaration, clinical trials could barely be conducted despite restrictions on patient care and the request that clinical trial personnel stay at home due to SARS-CoV-2 infection. This may be one reason why DCT spread differently in Japan than in Europe and the United States. Below, we discuss the regional differences in regulations and culture that might affect the implementation of DCT (Supplementary Datasheet 1, Supplementary Table 4, Step 4. 23, 24, Supplementary Table 5).

As shown in the interview results highlight the need to recognize the regional differences and cultural contexts of each country. In certain countries, including Japan, both physicians and patients tend to prefer face-to-face care to home healthcare and telemedicine (Supplementary Datasheet 1, Supplementary Table 5). Japan has limited experience with DCT; therefore, building upon small successes and aligning stakeholder mindsets during its implementation is critical in the initial stages. To motivate partner medical institutions and secure their understanding and cooperation, it is important to involve them with the central medical institution, which plays a key role in the DCT system within national projects, and to share information and experiences related to DCT (Supplementary Datasheet 1, Supplementary Table 5).

### 4.4 Regulations for DCT

Interviewees mentioned restrictions on the deployment of mobile nurses and certain tests and surgeries at local hospitals in certain countries or regions (21). They provided examples of PIs, nurses, and doctors in a van parked in a parking lot near the participant’s workplace or tent set up in a community parking lot, such as a church or school, to perform clinical trial functions. Whether such operations are possible depends on the regulations of each country or region.

In Japan, regulations concerning outsourcing under Article 39–2, Paragraph 23 of the Japanese Good Clinical Practice (J-GCP) guidelines constitute a significant barrier to DCT implementation (25). Specifically, when a medical institution outsources part of a clinical trial’s work, it must contract with the entity performing the outsourced tasks. In contrast, in the United States, sponsors can contract directly with nurses or physicians. Furthermore, there are no explicit regulatory provisions specifying the types of business operations or medical procedures that may be outsourced. It is generally understood that outsourcing the administration of injectable drugs, a medical procedure, is not permitted in Japan. However, regulatory authorities have since clarified that the administration of injectable drugs does not constitute a medical procedure, but is instead considered part of clinical trial procedures. As such, outsourcing of these procedures is permitted (unpublished data). Consequently, the administration of injectable drugs is now regarded as permissible within DCT frameworks, and a broader implementation of DCT in Japan is anticipated. On the other hand, the Worker Dispatch Law is a law that aims to protect temporary workers and ensure the proper operation of temporary staffing businesses. However, in the medical field in particular, cautious rules have already been established, and it is important to engage with regulatory authorities.

In our survey, regarding the delivery of investigational drugs to participants’ homes, many investigational drug managers responded that they expect to reduce their workload by adopting the “Depot to Patient” model, which involves directly delivering investigational drugs from the investigational drug warehouse to participants’ homes via medical institutions. However, responses from clinical trial office staff expressed skepticism regarding the effectiveness of this model in reducing workload. This discrepancy appears to stem from the interpretation of Japan’s GCP regulations, which state that even in a Depot to Patient scenario, the prescription and administration of investigational drugs remain the responsibility of the clinical trial site. This regulatory interpretation is identified as one of the key challenges in implementing the Depot to Patient model. This topic is discussed in Section 4.4 “Regulatory Considerations for DCT.” Additionally, the relevant literature on DCT for investigational drugs has been added to the reference list (26).

Considering these challenges facing DCT, regulatory authorities should support DCT through the establishment and revision of laws and regulations. Guidance documents specific to DCT have been issued in Switzerland (27) and Denmark (28), and the United States Federal Drug Administration has recently published draft guidelines on DCT. As the importance of global clinical trials in developing new drugs increases, each country is likely to develop guidance on DCT that includes specific methodologies based on its own regulations while considering global harmonization (Supplementary Datasheet 1, Supplementary Table 4, Step 4. 25).

### 4.5 IoT related issues

To promote the use of digital communication technology in healthcare, each hospital’s electronic medical record system should be linked to telemedicine, home nursing, written explanations, and consent. In Europe and the United States, compatibility between electronic medical record systems and other digital information has emerged as a concern. For instance, data access to electronic medical record systems from external sources and cloud-based electronic medical records shared with partner medical institutions are highly desirable. To achieve this, each IoT must have a sufficient level of security with safeguards regarding confidentiality, eliminating system inconsistencies, and allowing reliable data collection (29). As similar problems are likely to arise in Japan, regulators must present an appropriate approach before a significant disruption to numerous systems occurs.

To promote global drug development in Japan, an environment that standardizes IoT should be created along with constructing a platform that can run multiple operations at multiple medical institutions simultaneously (Supplementary Datasheet 1, Supplementary Table 5) (15, 16).

Monitoring technologies, including wearable and other electronic devices, are often utilized in DCT, along with the input and management of electronic records and communication via the Internet. This study identified the need for medical staff to have a high affinity for IoT, and securing these personnel increased the potential burden of CRC. Developing and introducing more affordable and user-friendly devices and systems is necessary to avoid rising labor costs in clinical trials. Furthermore, interviewees highlighted the importance of data reliability. The reliability of ePRO and the accuracy of non-medical wearable devices utilized in DCT are significant concerns and should be thoroughly addressed in the future (Supplementary Datasheet 1, Supplementary Table 4, STEP 4, 26).

### 4.6 The DCT cost

The interviewees indicated that costs increased in the early stages of DCT implementation because of the initial costs of system introduction and the organization of personnel structure (Supplementary Datasheet 1, Supplementary Table 4, STEP 4, 29). However, the costs associated with clinical trials can be reduced and reduced in the long term. In other words, implementing DCT is expected to promote case enrolment and shorten the recruitment period, while reducing costs for CROs, SMOs, and medical institutions by shortening the study period and reducing the number of hospital visits. In addition, cost reduction was anticipated by decreasing the labor hours required for data acquisition and DM. The total cost benefits of DCT implementation have not been calculated precisely currently, but will be further clarified by future experience with DCT.

### 4.7 Clinical trial design

The standardization of implementation plans and other documents anticipating the introduction of DCT should be considered (Supplementary Datasheet 1, Supplementary Table 4, STEP 4, 28). A simple example is preparing a schedule of clinical lab tests for major testing performed at an investigational site and at a nearby partner site. Designing and promoting trial designs incorporating DCT can expand the amount and quality of data collected while reducing or eliminating hesitancy regarding DCT. Because not all clinical trials can be conducted with a fully remote DCT, it is important to focus on areas where it can be used. For example, diagnosis using large equipment cannot be performed in home care, but follow-up care, collection of adverse event data, and obtaining patient diaries can all be implemented in the DCT (30).

The global promotion of DCT is expected to reduce the burden of travel and accommodation for pediatric patients and their families, while also accelerating the development of therapeutic drugs for patients who face difficulties leaving their homes. This includes individuals with rare diseases or psychiatric and neurological disorders that require long-term home-based care and populations that often face significant barriers to participating in clinical trials conducted at distant medical institutions. We believe that expanding the DCT will significantly advance drug development for these underserved patient groups by improving access to clinical trials.

As mentioned at the beginning, many papers have been written about DCT, but this paper presents a more positive perspective on DCT than previous studies (16, 29–31). However, there has been insufficient verification to demonstrate that DCT is equivalent to or superior to conventional clinical trials in terms of quality, speed, and cost. Future meta-analyses of numerous DCT studies are necessary to evaluate this (30, 31).

### 4.8 Ethical consideration

The purpose of this study was solely for internal reference, and participation was voluntary: no participant was required to report their decision to superiors. Although formal written consent was not obtained, each interviewee received a brief information sheet outlining the study objectives, data use, and confidentiality measures, and verbal agreement was documented prior to data collection. As the interviews did not involve patient personal information or medical data, no formal ethical review was required under Japanese MHLW guidelines (Ethical Guidelines for Medical and Biological Research Involving Human Subjects). Confidential data were handled in accordance with each company’s policy: transcripts and notes were stored encrypted on a cloud platform with detailed access logs, and physical download requests were limited to a standalone PC. Prior to commencement, we confirmed with each organization that the planned disclosures posed no issues.

### 4.9 Abstract nature of future applications and policy implications

Japanese regulations have not yet issued detailed, practical guidelines for the various aspects of decentralized clinical trials (DCT). Additionally, while the development and promotion of educational programs incorporating digital tools is recognized as important, the specifics of such programs have yet to be established. Therefore, it is desirable to accumulate experience with DCT in Japan, using our findings as a reference, and to develop practical procedures and educational initiatives. Technological innovations, such as integration of remote monitoring devices with electronic trial-management systems, mobile health applications for patient-reported outcomes, and IoT-enabled vital-sign trackers, should be piloted to streamline decentralized operations.

### 4.10 Strengths and limitations

Since the study is based on interviews conducted with multiple practitioners who have extensive experience in implementing DCT and consider the changes that occurred before and after the COVID-19 pandemic, it is more specific and comprehensive than the limited number of previously published reports based on experience. Additionally, the interviewees were responsible for development work and provided knowledgeable, positive, and practical insights regarding DCT. However, the analyzed information did not cover all the DCT cases. In this study, we adopted SCAT analysis to objectively analyze the interviews; however, it is difficult to say that our subjective views have been completely eliminated. Additionally, while we have published survey results on the current status of DCT implementation in Japan and globally (1) and in Japan specifically (26), obtaining information on the current status of DCT implementation and stakeholder opinions in regions including Japan that are planning to fully implement DCT in the future, considering the latest trends, remains a future research topic. It should be noted that comparisons with other regions are still in their early stages, and direct comparisons with specific Asian countries are beyond the scope of this paper. However, the approach of this paper, which focuses on “Asia,” is considered to be an important direction for future DCT research in Japan, including the Asian region. In China, South Korea, Taiwan, and India, the introduction, research, and expansion of strategic initiatives are being reported in the media.

## 5 Conclusion

To understand the current state of DCT, which has been promoted in Europe and the United States in recent years, we conducted interviews with individuals knowledgeable about DCT at pharmaceutical and medical device companies. We then analyzed and organized their responses using SCAT analysis. By comparing the situation before and after the coronavirus pandemic in Europe and the United States, we identified the issues associated with the active introduction of DCT.

The benefits of introducing DCT were evident; our results showed that DCT facilitated patient access to clinical trials, eliminated patient inconveniences, facilitated case enrolment, and improved the quality of the clinical trials. This study identified the following issues: local culture and regulations regarding home and telemedicine; the sharing of patient information, including medical records online, to those involved in clinical trials; the development of IT infrastructure; IT literacy among those involved in clinical trials and participants; and the costs and burdens associated with the introduction of DCT. This study provides useful information regarding the challenges facing regions, including Japan, that are promoting the introduction of DCT and will contribute to the global promotion of DCT. Going forward, we believe that gathering information on the implementation status of DCT and the perspectives of stakeholders in regions, including Japan, who are planning to introduce DCT in line with the latest trends will be an important area for further investigation.

## Data Availability

The original contributions presented in the study are included in the article/Supplementary material, further inquiries can be directed to the corresponding author.
