# Supplementary material for: Considerations on the implementation of DCT: a SCAT-based analysis of fact-finding interviews in Europe and the United States, with implications for regions newly adopting DCT, including Japan
Source: Front Med (Lausanne). 2025 Oct 31;12:1521135. doi: 10.3389/fmed.2025.1521135 (PMC12616372; doi:10.3389/fmed.2025.1521135)
Supplement: Supplementary file 1 [file Data_Sheet_1.pdf]

Supplementary Table 1 A.SCAT analysis on DCT implementation in Europe and the US before the COVID-19 pandemic

	Text	Step 1. Notable words and phrases in the text	Step 2. Paraphrasing textual phrases	Step 3. Extra-textual concepts that would explain Step 2	Step 4. Themes, compositional concepts	Step 5. Questions or issues
1	DCT has just begun to be incorporated into clinical studies as a new idea, but there was little experience.<br><br>In addition, they were not aggressive in their introduction.	New Ideas	Although DCT has been initiated and will be introduced, the specifics of its implementation are unclear.	DCT was a new concept and was not yet widespread. Little experience exists with these trials.	DCT was a newer, innovative concept. Medical facilities and physicians were originally concerned about introducing the trial method as they had little experience in operating it.	How to proceed with unfamiliar DCT
2	Remote consent acquisition and data monitoring had been implemented in some areas but were not as widespread.	Remote consent acquisition and data monitoring	Individual IoT used in DCT was not yet widespread.<br><br>Many stakeholders need to be briefed on IoT.	Physicians with some affinity for IoTs obtained consent remotely. IoT was mainly used for consent acquisition and data monitoring.	A few physicians were willing to adopt some of the DCT functions, such as to obtain consent, to monitor data, and other aspects of patient care; however, that willingness was not universal.	How do we get physicians interested?<br><br>How can we get physicians to adopt IoT into their practices?
3	In a few cases, it was necessary to explain the safety and precautionary measures against data leaks in DCT to ethics review committees and others to convince them that it was feasible.	Methods that can be appropriately implemented	The ethics review committees must certify that there are no serious problems.	The ethics review committees required detailed explanations of the appropriateness of the DCT.	The ethics review committees required a detailed explanation of how the DCT would operate, including appropriateness, and committee members expressed concerns about whether data could be accessed from outside the institution.	Ethics committees tend to be cautious about new methodologies, perhaps because of the potential impact on privacy laws.

4	Remote data entry into EDC to electronic medical records was possible in some facilities.	EDC, Electronic medical records, and remote data entry	Data entry to EDC and electronic medical records was possible in certain institutions, and they were validated and verified. Views on IoT varied by the medical institutions	External access to medical record data was allowed under contract in some institutions in the US, especially not in Europe.	External access to medical records was possible at certain institutes. Interviewees mentioned concerns regarding how stakeholders, including sponsors, facilities, and partner institutions, would share the costs of introducing and implementing DCT in clinical trials.	About filling in many types of electronic medical records and worksheets and their security, the reality of external access to medical records, which is considered difficult, how to output facility data, and how the Japanese electronic medical record system is not the most up-to-date system. Remoteness may be difficult, but not impossible
5	Certain institutions did not allow remote access to the medical records because they did not have sufficient systems.	Certain institutions did not have adequate systems for remote access to medical records.	Differences in views on IoTs among medical institutions.	Cases in which explanations of how to use the data and ensure data security were required when institutions did not accept remote monitoring of patient data and medication compliance, though technically possible, because of resistance to data access from outside. Special diagnostic imaging. could not be performed remotely.	Many factors inhibited the introduction of DCT, such as the lack of robust system at the medical facilities.	How can we close the gap in medical facilities’ ways of thinking about IoT?

6	Licenses required by each state in the US were handled by assigning nurses who held licenses in the state where the study was conducted and having them work under the direction of a physician.	State licensing, physician's orders	To perform DCT in the US, medical professionals are required to have a state-specific license.	Licensing by state is unique in the US. The difference between Japan and the US is that medical professionals do not have a contract with a facility. Nurses are not affiliated with institutions but with companies or CROs that provide services.	Nurses are licensed by each European country and state in the US. Nurses were assigned in each country or state and had to perform their duties under the direction of a physician.	Home medical care, including the administration of investigational drugs, would be a hurdle in Japan.
7	Pharmaceutical companies have surveyed and consulted on DCT trials.	DCT consideration by pharmaceutical companies	Study the feasibility of DCT in terms of both digital and procedural aspects.	COVID-19 has made a significant step forward for DCTs. In particular, the new world of clinical trials brought about by online medical care was an unknown and inexperienced field, and the benefits were unclear. However, the times were moving towards the introduction of DCT.	This study highlighted the importance of the medical environment, including regulations, digital tools, and the establishment and documentation of decentralised procedures in clinical trials.	The current state of information technology in Japan and the strictness of the Personal Information Protection Law makes it difficult to access electronic medical record data externally.

Abbreviations: COVID-19 (the novel coronavirus disease), CRO (Clinical Research Organization), DCT (Decentralized Clinical Trial), EDC (Electronic Data Capture), IoT (Internet of Things), SCAT (Steps for Coding and Theorisation), US (United States)

Supplementary Table 2 B.SCAT analysis on DCT implementation in Europe and the US after the COVID-19 pandemic

	Text	Step 1. Notable words and phrases in the text	Step 2. Paraphrasing textual phrases	Step 3. Extra-textual concepts that would explain Step 2	Step 4. Themes, compositional concepts	Step 5. Questions or issues
8	Physicians have become accustomed to being forced to implement telehealth when patients cannot come to the8.	Telehealth implementation	Unable to be treated in a medical facility	Lockdown, clinical trials cannot be conducted.	The global spread of the novel coronavirus (COVID-19) has significantly impeded the promotion of clinical trials and the submission of product applications for approval. The lockdown caused by the spread of the COVID-19 infection prevented patients from visiting institutions, forcing them to implement DCT to proceed with clinical trials.	If many examples are collected, the concept will change. It is necessary to collect best practices.
9	Vendors offering systems for DCT also emerged. Providing systems that are easy to use has facilitated the introduction of DCT.	DCT vendors, easy to utilise systems	The emergence of specialised vendors and the introduction of new technologies promoted DCT.	With the various developments in medical records vendors, several vendors propose systems that take advantage of importing test data into the medical record and facilitate its use.	In parallel with the development of DCT systems, institutional implementation frameworks and procedures were established, and vendors offering DCT systems emerged.	Has the emergence of vendors been able to incorporate stakeholders who have less affinity for IoT?
10	Only approximately 5 out of 50–70 facilities were willing to conduct	Over 70% of facilities allow	Medical facilities have become almost acceptable	Remote monitoring is available at many facilities. It was available at certain	Remote monitoring is now a viable option at almost all facilities.	Methods of remote monitoring need to be considered. For example, is it possible to access the

	remote monitoring in the past. However, now more than 70% of facilities conduct remote monitoring.	remote monitoring.	for remote monitoring.	facilities before the pandemic, but after COVID-19 spread, remote monitoring was accepted without any problems.		electronic medical record externally and match it to the CRF?
11	The mindset of those involved has changed regarding the importance of proceeding with necessary treatments and trials, even during the COVID-19 pandemic, and cooperation in conducting clinical trials has been obtained.	Mindset of stakeholders.	Mindset toward DCT implementation has changed.	Actively adopt DCT as a means to achieve clinical trial goals.	The mindset changed for proceeding with clinical trials by introducing DCT, and positively considering proceeding with DCT accelerated its introduction.	Further analysis of factors that led to the change of mindset.
12	Most US facilities now have external access to data. Europe remains challenging.	US allows external data access, Europe difficult.	External data accessibility still varies by country.	Access to medical record data from external sources varies by country, but it is almost always possible in the US.	The need to conduct trials without monitors visiting the facility has resulted in most facilities in the US now having external access to data.	Need for verification of the information, security and privacy protection support.
13	While on-site training for physicians and others on the protocol and devices is difficult to implement, devices, simulators are sent to facilities, and	On-site training, VR training	Pre-use training on medical devices can now also be provided using VR and other methods.	Certain medical devices require training in their use. Training for physicians was conducted hands-on before the pandemic. This is an essential point in situations	Pre-use training for medical professionals on medical devices can now be done externally using virtual reality and other technologies.	Difficulty in on-site training

	training is provided via VR.			where hands-on training is not available.		

Abbreviations: COVID-19 (the novel coronavirus disease), CRF (Case Report Form), DCT (Decentralised Clinical Trial), IoT (Internet of Things), SCAT (Steps for Coding and Theorisation), US (United States), VR (Virtual Reality)

Supplementary Table 3 C.SCAT analysis on advantages and disadvantages of DCT implementation

	Text	Step 1. Notable words and phrases in the text	Step 2. Paraphrasing textual phrases	Step 3. Extra-textual concepts that would explain Step 2	Step 4. Themes, compositional concepts	Step 5. Questions or issues
14	Patients who could not participate in the clinical trial due to distance from the trial site will be able to participate, and enrolment of participants will become easier.	Benefits: Patients who were previously unable to participate can participate in clinical trials, making enrolling easier.	Easier access for distant patients.	Entry of patients from widespread areas outside the vicinity of the medical institution is possible, increasing the diversity of the participants.	Introducing DCT enables patients in remote areas who struggled to participate in conventional clinical trials to access treatment. This approach facilitates greater participation in clinical trials, thereby increasing access and expanding the diversity of the participants.	A comparison of the time needed for the enrolment needs to be made.
15	Including eligible patients in the study in less time than it took on-site is now possible.	Eligible patients have a shorter time frame.	DCT allows the enrolment of eligible patients in a shorter timeframe.	Inclusion has accelerated because it is possible to recruit a wide range of patients, but further confirmation is needed as to why eligible patients can be included quickly.	DCT can reduce the travel burden and some of patient inconvenience while providing potentially more inclusive, comfortable environment for subjects. For example, the core facility can remotely monitor patients during visits, which previously required hours of observation, and acquire data in real-time to confirm patient safety via videoconferencing. In other words, the burden of patient travel and waiting times can be reduced.	It is necessary to ascertain why eligible patients can be included in a DCT in a short period of time.
16	Whereas it used to take hours for a single patient to come in for a single visit, now the core facility can use technology to check up remotely, get continuous data, and contact patients via teleconference, to	Remote consultation and teleconferencing with patients previously took hours for each visit.	DCT saves time in clinical trials (remote checking, teleconferencing, patient data entry).	Patient inconvenience is reduced.		Wouldn't it reduce the motivation of the partner medical institutions?

	make sure they are safe and doing what they need to do.					
17	Patients who could not participate can now participate in the trial, but patient and follow-up compliance has improved.	Improved compliance.	The quality of the clinical trial is improved.	DCT will eliminate the need for patients to visit a facility, improving compliance by eliminating the need for them to travel to a facility far from their homes regularly.	DCT facilitates follow-up and improves patient compliance. In addition, implementing remote clinical trial procedures has increased efficiency and flexibility, including how clinical trials are conducted. In addition, DCT allows data collection independent of visits, which is expected to reduce the amount of missing data and improve the quality of clinical trials.	Consideration of how to advertise the benefits of DCT.
18	All physicians, medical staff, and patients involved in the trial must be familiar with digital technology.	Affinity for digital technology.	Not knowing how to use digital technology may limit participation in DCT.	Operationalising DCT requires that all those involved in the DCT be comfortable with digital technology. For example, lack of access to the technology or not knowing how to use it may limit participation in DCT.	All physicians, medical staff, and patients involved in the clinical trials in question must demonstrate ability to learn and use digital technology. For instance, a lack of access to technology or an inability to utilise it may restrict participation in a DCT.	Operationalising DCT requires all those involved in all DCT to be strong in digital technology.
19	It may be difficult to check the accuracy of patients' self-entered data, e.g., whether patients understand	Patient self-entry of their data makes it difficult to ensure	Data quality depends on the patient's	Ensuring quality regarding DCT is difficult, especially in remote clinical trials.	Collecting patient information through wearables and other devices requires patients to understand the process and	Responding to increased regulation of IT systems based on data reliability.

	the data they are entering, making it more difficult to ensure the data quality.	accuracy and data quality.	understanding of the data.		be actively involved in the clinical trial. Without patient understanding and cooperation, it is difficult to verify the accuracy of the data entered and to ensure integrity and reliability. In addition, the possibility of continuous data collection via devices may increase measurement error.	
20	Patients enter data at home, making it difficult to verify the accuracy of the data.	Data accuracy.	Difficulty in confirming the accuracy of data.	It is difficult to ensure the accuracy and quality of data.		How will the accuracy of data be verified?
21	Although it depends on the type of study, it is believed that the quality of studies is improving at the data collection step. It will be important to consider the collection of data for the primary endpoint when, for example, echocardiography or other tests should be performed under the same conditions.	Improved quality of trials and test data collection under the same conditions.	Need to improve the quality of the study, testing, and other conditions.	There is an advantage in reducing the number of missing data because follow-up data can be obtained regardless of the hospital visit. However, when imaging conditions and equipment performance, such as image data, are greatly affected or when the skill of the photographer affects the data, it is necessary to arrange the conditions or bring in experts, which is a limitation of DCT.	It may be difficult to adapt to situations in which data may be affected by testing equipment and conditions, and the condition settings need to be studied in detail. Since there are multiple facilities, testing and other conditions need to be unified.	To evaluate the quality of data obtained in DCT, is it not necessary to compare them among the same study?
22	Payments to facilities are the same as before, and in some cases, additional costs associated with	Payments to facilities, costs associated with conducting trials	Virtual costs are added to normal costs, short-term costs are high, and shortening the	At the time of introduction, there are additional costs for capital investment, additional equipment,	The clinical trial operating costs may increase as new systems and structures are developed and implemented.	To evaluate the value of DCT in its true sense, we need to investigate cost and

	conducting trials virtually, such as Wi-Fi usage fees, are required, so, at this point, cost savings have not yet been achieved.	virtually, and cost savings.	clinical trial period is expected to reduce long-term costs.	communication costs, making the cost high. Further, it is necessary to investigate whether DCT can reduce costs and determine when it will become a steady-state system.		time savings changes over time.

Abbreviations: DCT (Decentralised Clinical Trial), IT (Information technology), SCAT (Steps for Coding and Theorisation)

Supplementary Table 4 D.SCAT analysis on challenges in introducing DCT

	Text	Step 1. Notable words and phrases in the text	Step 2. Paraphrasing textual phrases	Step 3. Extra-textual concepts that would explain Step 2	Step 4. Themes, compositional concepts	Step 5. Questions or issues
23	The culture in China does not favour home health care, and neither do the patients. Their conservative nature and aversion to change can be problematic.	China is a culture that does not favour home health care and is conservative in nature.	Cultural and regional differences need to be considered for DCT operations. Consensus and mindset of all stakeholders are important. Since Japan has a unique culture and mindset, the cultural factor could be a point to consider when introducing standards from other countries to Japan.	Cultural differences between countries should be noted. For example, some cultures do not favour home care, telemedicine, or direct-to-patient delivery of investigational drugs, and healthcare professionals and patients may not choose DCT.	Being aware of the regional culture is necessary when implementing DCT. For example, there are countries, including Japan, where direct communication with the primary care physician is important for patients. Patients in China do not favour home care, telemedicine, delivery of drugs to patients. Neither healthcare professionals nor patients may prefer DCT. A conservative disposition or reluctance to embrace change is another obstacle.	The cultural and regional differences need to be examined.
24	A few healthcare professionals believe that interactions should be done face-to-face, which hinders home health and direct-to-patient elements in DCT.	A culture that believes interactions should be in face-to-face form.	Physicians insist on face-to-face care and resistance to new things for clinical trials.	Resistance to new things is a major challenge, especially in Japan's busy medical field. Medical professionals are extremely sensitive (and cannot afford) the time and resources required to introduce new things, which can slow down normal work. At the same	The prevailing culture among healthcare providers suggests that clinical trials should be conducted face-to-face. In other words, consensus and a shared mindset among all stakeholders are crucial for the success of DCT implementation.	How can cultural norms be dispelled?

				time, there is a conservative mindset and reluctance to embrace DCT among healthcare professionals, who see the introduction of dynamic and innovative change as a risk. In other words, consensus and the mindset of all stakeholders are important.		
25	One challenge in global clinical trials is that resources are required when many things need to be addressed in each country, such as significant differences in regulations and practices. From country to country, which could be a barrier to implementation. For example, regulations for mobile nurses and home health nursing.	Resource requirements for global clinical trials, regulation on mobile nurses, and home health nursing.	Medical disparities by region, home health nursing, and inability to perform procedures at local centres.	Clinical research team leaders have created the appropriate environment to deploy DCT through global vendors but hesitate for clinical research in specific countries, which has led to regional differences in the adoption of DCT. Regional differences are a fraudulent factor in advancing DCT.	Many practical regulatory differences need to be addressed from country to country, such as mobile nurse and home health nursing regulations, which require resources and can also be a barrier to DCT implementation. For example, Canadian regulations do not allow mobile nurses, and French regulations may not allow the use of eConsent electronic signatures due to privacy concerns.	Substantial country-specific differences in regulations and medical practices can be a barrier to the introduction of DCT in global trials. We need to investigate the situation in other countries and consider how Japan can harmonise with the rest of the world to avoid Japan being passed by.
26	There is the issue of the digital divide. All parties involved in the study, including patients, physicians, and other staff, must adapt to the IoT. The decision to	The digital divide issue.	Need to consider in advance whether DCT can be performed due to age and adaptability to IoT.	Since ePRO and other inputs must be performed by the subject, IoT affinity, age, and other factors must be considered to determine whether the	There is the issue of the digital divide (IT literacy and adoption). All study personnel, including patients, physicians, and staff, must be willing to adapt to IoT. Their affinity	Measures against digital deviations and measures for those who are not good at IT, such as older adults, are important.

	implement DCT must be based on the age of the patients and physicians and their affinity for IoT.			patient can participate in the study. Patients and all parties involved have an affinity for IoT. In addition, if DCT cannot be performed, the burden on the support department will increase.	for IoTs and patients' age will determine whether or not they can conduct a DCT. In particular, trials involving older adult patients require attentive technical support, which might burden clinical research coordinators (CRCs). However, there are insufficient measures to deal with the increased burden on CRCs. Furthermore, patient education and periodic checks are essential to ensure the quality of data input by patients themselves. Securing digital human resources at each implementing medical institution is crucial.	
27	Data privacy must be ensured through various data transmission and reception methods.	Various methods of sending and receiving data ensure privacy.	Protecting personal information, ensuring privacy, and data validation (systems that comply with regulations) will be necessary.	Data security is important from the standpoint of personal information protection.	While there are various data transmission methods and platforms for data acquisition, it is paramount to ensure the protection of personal information, privacy, and data validation (a regulatory-complaint system). The challenge, however, lies in protecting patients' personal information and improving the quality of clinical trials at the facility level. Consequently, it is recommended that small-scale pilot studies, such as the one conducted in the US,	Establishment of IT systems that ensure data security and quality.

					be conducted in Japan to determine the optimal method of application to be used.	
28	In a few cases, there is resistance to accepting trial designs with cutting-edge elements, medical professionals continue to use traditional methods.	Study design with cutting-edge elements continues to use traditional methods.	Reforming the existing implementation plan will take time.	To efficiently implement DCT, the trial design needs to be significantly transformed for DCT.	The traditional study protocol must be significantly modified, which delays the finalization of the protocol and the initiation of clinical trials. Those responsible for conducting clinical trials, including physicians and sponsors accustomed to conventional study protocols, often believe that conventional methods lead to the early termination of clinical trials.<br>Moreover, regional disparities may serve as deterrents to DCT implementation. Healthcare professionals may possess a conservative mindset, leading to reluctance in accepting DCT. They may perceive the adoption of dynamic and	Need to create a new implementation plan template.

					innovative changes as risky.	
29	Resource issues with the increased burden of contracts and contract writing that clinical research teams must enter into to implement DCT. This has increased the burden on support departments, CROs, and CRCs, as they must prepare complicated procedures and other tasks, including vendor monitoring.	Resource issues due to increased contracts led by the introduction of DCT.	More contracts and human resources mean the huge costs and personnel are required; FDA and EMA require the clients to ensure that the testing personnel, including contractors, are qualified and experienced in DCT work.	One of the disadvantages of DCT implementation is the increased workload and the accompanying human resources required. It requires substantial cost and many personnel.	When DCT is newly introduced, contracts and procedure manuals must be concluded for each medical institution, which requires huge costs and personnel. Furthermore, FDA and EMA require sponsors to confirm that study personnel, including contractors, are qualified to perform DCT work. This requires complicated procedures, including vendor monitoring, and increases the burden on support departments, CRO, and CRC.	Increased cost issues when introducing DCT.

Abbreviations: CRC (Clinical Research Coordinator), CRO (Clinical Research Organisation), DCT (Decentralised Clinical Trial), eConsent (Electronic Informed Consent), EMA (European Medicines Agency), ePRO (electronic Patient Reported Outcome), FDA (Food and Drug Administration), IoT (Internet of Things), IT (Information Technology), SCAT (Steps for Coding and Theorisation), US (United States)

Supplementary Table5 Theoretical explanation and Issues to be pursued

**Theoretical explanation**	Regarding the situation before the COVID-19 pandemic, DCT was still a new concept, and medical facilities and physicians were not actively involved in its introduction.
	After the COVID-19 pandemic began, patients could not come to the hospital, and DCT had to be introduced. The system and procedures were developed, and vendors offering DCT and VCT systems emerged, accelerating the introduction of DCTs.
	The introduction of DCT will enable patients in remote areas who would otherwise have difficulty participating in conventional trials to participate in clinical trials and gain access to high-quality, state-of-the-art medical care.
	All physicians, medical staff, and patients involved in the clinical trial must have an affinity for digital technology.
	There is a digital divide problem. All study personnel, including patients, physicians, and staff, must be adaptable to the IoT, and a decision on whether or not to implement DCT will be necessary, depending on age and affinity for the IoT.
	While there are various data transmission methods and platforms for data acquisition, privacy protection, privacy assurance, and data validation (a regulatory-compliant system) are important, and the challenge is how to ensure the protection of patients' personal information and the quality of clinical trials at each site.
	The number and size of organisations that can implement DCT are limited; as a result, contracts and procedure manuals must be concluded for each medical institution when introducing DCT, which entails enormous costs and a large number of personnel.
**Issues to be pursued**	Japan is conservative or does not want to change, and some countries hesitate to deploy DCT even if the infrastructure is in place.
	In certain countries, the culture does not favour home medical care; as a result, patients do not choose it. The challenges in each region are disincentive for clients to proceed to DCT.
	A few healthcare providers continue to use traditional methods because of a culture that sees them as relative to face-to-face forms and resistance to accepting the study design. In other words, there are significant cultural challenges.
	How do we get physicians interested?<br>How can we change their mindset?

	Data accuracy must be conducted remotely, and the quality must be ensured and proven (DCT vs. traditional clinical trials).
	Establishment of infrastructure and IT systems on the facility side<br>Preparation of a new implementation plan template.
	Privacy and cybersecurity are inseparable issues for DCT.
	In Japan, there seems to be sensitivity to non-compliance and deviations and resistance to pointing them out to physicians.

Abbreviations: COVID-19 (the novel coronavirus disease), DCT (Decentralised Clinical Trial), IoT (Internet of Things), IT (Information Technology), VCT (Virtual Clinical Trial)
