# Supplementary material for: Considerations on the implementation of DCT: a SCAT-based analysis of fact-finding interviews in Europe and the United States, with implications for regions newly adopting DCT, including Japan
Source: Front Med (Lausanne). 2025 Oct 31;12:1521135. doi: 10.3389/fmed.2025.1521135 (PMC12616372; doi:10.3389/fmed.2025.1521135)
Supplement: Supplementary file 2 [file Data_Sheet_2.pdf]

Supplementary Figure 1

Questionnaires for the Interview on Fact-Finding Research on DCT

1. Backgrounds: We have been rewarded the Japan Agency for Medical Research and Development (AMED) grant for the research "Study on issues and solution of Decentralized Trial in Japan and overseas." The overall objective of this research is to understand the current status of Decentralized Clinical Trials (DCTs) (Virtual Clinical Trials (VCTs)), which has been a hot topic in recent years, identify issues, and make recommendations to the government for the promotion of DCTs in Japan. The interview contents will be published as a part of a research report to AMED. Still, we will let you know the contents to check before submitting and modify it if needed. It would be appreciated if we could have a discussion frankly.

2. Objective: To investigate the actual status of DCT (Decentralized Clinical Trials/Virtual Clinical Trials) for overseas clinical trials of diseases other than COVID-19, changes before and after the spread of COVID-19 infection, and differences between Japan and the United States.

3. Methods
   1) Interviewees: Personnal of pharmaceutical companies and medical dvice companies or CRO involved in implementation of DCT.
   2) Method: Interview the following contents via web meeting
      ① Status of DCT implementation before and after the spread of COVID-19 infection
         ✓ Status of DCT implementation <u>before</u> the spread of COVID-19 infection
           What exactly do you conduct clinical trials with DCT such as IC, Non-visiting monitoring, Follow-up Medical checkup (blood collection and taking EKG by visiting patient's house, Web-based interview with a doctor, and etc.)?
           Did you cooperate with a particular vendor, such as CRO, System Vendor, etc., in the introduction of DCT? About what did you consult the vendors?
         ✓ Status of DCT implementation <u>after</u> the spread of COVID-19 infection
           What exactly do you conduct clinical trials with DCT such as IC, Non-visiting monitoring, Follow-up Medical checkup (blood collection and taking EKG by visiting patient's house, Web-based interview with a doctor, and etc.)?

Do you cooperate with a particular vendor, such as CRO, System Vendor, etc., in the introduction of DCT? About what do you consult the vendors?

- ✓ Points that have changed significantly before and after the spread of COVID-19 infection (scope of DCT, framework, measurement devices, IoMT/IoHT)
- ✓ Items need to introduce DCT in the future
- ✓ Comparison between Japan and the U.S. regarding the above (if possible)

② *Pros and cons* of introducing DCT

- ✓ *Pros*
- ✓ *Cons*, issues: *eg.* framework construction, reliability
- ✓ Others

③ Requests for regulations of DCT, *etc.*

4. Others
