# Supplementary material for: Considerations on the implementation of DCT: a SCAT-based analysis of fact-finding interviews in Europe and the United States, with implications for regions newly adopting DCT, including Japan
Source: Front Med (Lausanne). 2025 Oct 31;12:1521135. doi: 10.3389/fmed.2025.1521135 (PMC12616372; doi:10.3389/fmed.2025.1521135)
Supplement: Supplementary file 3 [file Data_Sheet_3.pdf]

Figure 2 Interview Guide (DCT Questions)

- What was the status of DCT adoption in Europe and the United States before the COVID-19 pandemic?
- What was the status of DCT adoption during and after the COVID-19 pandemic?
- What are the advantages and disadvantages of DCT implementation?
- What challenges are associated with introducing DCT?
- How are DCT-based clinical trials conducted?
- What is the overall landscape of DCT in each region (Europe and the United States)?
- What is each company’s approach to DCT?
- What is the current regulatory status of DCT?
- How are companies engaging with regulatory authorities on DCT-related issues?
- How are the costs of DCT trials (including government subsidies) allocated?
- How reliable are the data generated through DCT?
- What are some success stories related to each company's DCT initiatives?
- What difficulties were encountered during DCT implementation?
- What solutions or mitigation strategies have been effective in addressing DCT-related challenges?
- Which disease areas are particularly well-suited to DCT?
